# Epidemiological Profile and *rpoB* Gene Mutations in Pulmonary Tuberculosis Patients From Sulaymaniyah, Iraq: Evidence of Novel Genetic Variations and Beijing Lineage Emergence

**DOI:** 10.1155/ijm/6691977

**Published:** 2025-10-14

**Authors:** Sirwan Mohammed Radha, Dana Khdr Sabir, Sirwan M. Mohammed

**Affiliations:** ^1^Department of Medical Laboratory Science, College of Science, Charmo University, Chamchamal, Kurdistan Region, Iraq; ^2^Department of Pharmaceutical Chemistry, College of Science, Charmo University, Chamchamal, Kurdistan Region, Iraq; ^3^Department of Biology, College of Science, University of Sulaimani, Sulaymaniyah, Kurdistan Region, Iraq

**Keywords:** Beijing strain, Iraq, multiplex PCR, *Mycobacterium tuberculosis*, *rpoB*

## Abstract

Tuberculosis (TB), caused by *Mycobacterium tuberculosis*, remains a significant global public health concern, including in Iraq. This study investigated the epidemiology, molecular characteristics, and rifampicin resistance–associated mutations of pulmonary TB in Sulaymaniyah, Iraq, from May 2024 to May 2025. A total of 77 confirmed pulmonary TB cases were identified, yielding an incidence rate of 9.09 per 100,000 inhabitants. This rate is notably lower than those reported in other Iraqi cities and may reflect a regional, and possibly national, decline in TB burden. Contrary to global trends, females comprised the majority of cases (*n* = 47, 61%). The highest prevalence of TB was observed among individuals aged ≥ 60 years, which may be attributed to immunosenescence and waning protection from the BCG vaccine. Monthly case distribution indicated seasonal variation, peaking between October and March. Molecular genotyping of 50 clinical samples revealed predominantly non-Beijing strains (98%), with the first molecular evidence of Beijing lineage in Iraq, indicating potential cross-border transmission. Analysis of the rifampicin resistance–determining region (RRDR) of the *rpoB* gene in 22 samples revealed no resistance-associated mutations, but detected several synonymous and novel nonsynonymous mutations outside the canonical RRDR. These genetic variations may represent early evolutionary adaptations without current resistance implications. Limitations in surveillance and diagnostic capacity may contribute to underreporting and obscure the true TB burden. This study highlights the evolving molecular epidemiology of TB in Sulaymaniyah, emphasizing the importance of ongoing molecular surveillance to inform public health strategies and control efforts.

## 1. Introduction

Tuberculosis (TB), caused by members of the *Mycobacterium tuberculosis* (MTB) complex of acid-fast bacilli, remains one of the most devastating infectious diseases worldwide [1]. It is among the leading causes of death from a single infectious agent, surpassing even HIV/AIDS in certain regions [2]. According to the World Health Organization's Global Tuberculosis Report 2024, an estimated 10.6 million people developed TB in 2023, resulting in 1.3 million deaths among HIV-negative individuals and an additional 167,000 deaths among those living with HIV [3]. Furthermore, it is estimated that approximately one-quarter of the global population harbors latent MTB infection [4]. Without appropriate treatment, TB may have a case fatality rate approaching 50% [5]. However, timely diagnosis and adherence to WHO-recommended therapy can greatly improve outcomes. This therapy, consisting of first-line anti-TB drugs over 4–6 months, enables up to 85% of patients to achieve successful treatment [6].

The standard regimen for TB includes four first-line agents: rifampicin, isoniazid, pyrazinamide, and ethambutol, with streptomycin occasionally included as a fifth agent [7]. Resistance to these drugs is mediated by mutations in key genes, including *katG*, *inhA*, *aphC*, and *kasA* for isoniazid, *rpoB* for rifampicin, *rpsL* and *rrs* for streptomycin, *embB* for ethambutol, and *pncA* for pyrazinamide [3]. Rifampicin is among the most potent anti-TB medications due to its efficacy against both actively metabolizing and slow-metabolizing bacilli, making it an essential component of first-line therapy for drug-sensitive TB [8–11]. In MTB, rifampicin binds the beta-subunit of RNA polymerase, inhibiting mRNA elongation. Resistance to rifampicin is primarily associated with mutations in codons 507–533 of the *rpoB* gene, known as the rifampicin resistance-determining region (RRDR), which accounts for approximately 96% of resistant cases [8, 9, 12]. Understanding mutations in this region is crucial for monitoring drug resistance and guiding treatment strategies. It has been estimated that approximately 580,000 new cases and 230,000 deaths globally are attributed to multidrug resistant (MRDR) TB annually [13].

A particularly concerning genotype of MTB is the Beijing strain which has been associated with increased virulence, transmissibility, and a higher likelihood of drug resistance [14]. This lineage has shown a remarkable capacity for transnational dissemination and has been implicated in outbreaks across multiple continents [15, 16]. Other studies reported that treatment failure is more common with the Beijing strain [15].

This study presents 1-year TB epidemiological data from May 2024 to May 2025 in Sulaymaniyah, Iraq. It also reports the first molecular identification of the Beijing genotype in the country and provides new insights into *rpoB* gene mutations among local MTB isolates.

## 2. Materials and Methods

### 2.1. Sample Collection

Demographic data of TB patients, including gender and age, were collected. For each patient, a pulmonary sample (sputum or bronchoalveolar lavage [BAL]) was obtained together with the date of collection. All samples were collected at the Consultation Chest and Respiratory Disease Center, Harem Private Hospital, and Anwar Sheikha Private Hospital in Sulaymaniyah, Iraq, between May 2024 and May 2025.

### 2.2. DNA Extraction

Initial detection of TB-positive patients was performed using direct microscopic examination with *Ziehl-Neelsen staining*, followed by molecular confirmation with GeneXpert MTB/RIF (Cepheid) and real-time PCR. Total mycobacterial DNA was extracted directly from pulmonary samples using Pathodetect-MTB Detection Kit (Mylab Discovery Solutions, Pune, India).

### 2.3. Multiplex PCR for Beijing Lineage Identification

Multiplex PCR was used for the detection of Beijing lineage in a total reaction volume of 25 *μ*L, containing 10 *μ*L of HotStarTaq Master Mix (BIO South Korea), 10 *μ*L of extracted DNA template, and primers (2 *μ*L forward primer, 2 *μ*L Reverse I, and 1 *μ*L Reverse II [Table 1]) [17]. DNA amplification was performed using touchdown PCR program under the following conditions: an initial denaturation at 96°C for 2 min; 10 cycles of denaturation at 95°C for 20 s, annealing at 66°C for 20 s, and extension at 72°C for 30 s; followed by 20 cycles of denaturation at 95°C for 20 s, annealing at 62°C for 20 s, and extension at 72°C for 20 s; and a final extension 72°C for 3 min.

### 2.4. PCR Amplification of the *rpoB* Gene

A forward and a reverse primer were used to amplify a 650 bp fragment of the *rpoB* gene flanking the RRDR (Codons 507–533) (Table 1). This region was targeted because it accounts for nearly 96% of rifampicin resistance, with Codons 526 and 531 being the most frequently mutated [8, 9, 12]. Primers were designed using the NCBI Primer-BLAST tool based on the standard genome of MTB H37Rv (Accession Number: NC_000962.3). PCR reactions were performed in single-cell PCR tubes using a Bio-Rad thermal cycler (CFX Manager, United States). Each reaction was carried out in a total volume of 24 *μ*L, comprising 10 *μ*L of total DNA template, 10 *μ*L of HotStarTaq Master Mix (ADD BIO, South Korea), and 4 *μ*L of primers (2 *μ*L each of forward and reverse primer). PCR cycling conditions were as follows: initial denaturation at 95°C for 5 min, followed by 30 cycles of denaturation at 95°C for 30 s, annealing at 56°C for 20 s, extension at 72°C for 35 s, and a final extension at 72°C for 4 min.

### 2.5. Agarose Gel Electrophoresis

PCR products of *rpoB* and *Rv0697c* were separated on 1.5% agarose gels stained with Safe Dye. Electrophoresis was carried out at 80 V for 1 h using Mupid One system (Japan), and bands were visualized with a gel imaging system (SCOPE 21, OPTIMA, Japan).

### 2.6. *Rv0679c* and *rpoB* Sequencing

The PCR products were sequenced using Sanger DNA sequencing (Macrogen, South Korea), and the resulting DNA sequences were analyzed using Chromas software, Version 2.2.6.

### 2.7. Results and Discussions: Epidemiology

During the 1-year study period, 77 confirmed cases of pulmonary TB were identified. Of these, 67 samples were collected from the Consultation Chest and Respiratory Disease Center in Sulaymaniyah city, while the remaining 10 were obtained from Harem Private Hospital and Anwar Sheikha Private Hospital. Based on the estimated 2025 population of Sulaymaniyah city (847,000) [18], the incidence rate of pulmonary TB was calculated to be 9.09 cases per 100,000 inhabitants (95% confidence interval: 7.06–11.12), which is lower than the rates reported in other Iraqi cities [19]. In 2010, surveillance data recorded 530 new and relapsed TB cases in Sulaymaniyah, corresponding to an incidence of approximately 31 per 100,000 [20]. Similarly, a 5-year study in Duhok Governorate reported a decline in TB incidence from 14.06 per 100,000 in 2014 to 10.34 per 100,000 in 2018 [21]. In contrast, Erbil city experienced a steady increase in incidence, rising from 16 cases per 100,000 in 2012 to 21.7 per 100,000 in 2016 [22]. At the national level, TB incidence has demonstrated a consistent decline over the past decade, decreasing from 45 cases per 100,000 individuals in 2013 to 21 per 100,000 in 2023 [19].

While the low incidence rate reported in this study may suggest encouraging progress—possibly due to broader BCG vaccination coverage, improved case detection, and increased public awareness—these results should be interpreted with caution. Sulaymaniyah is one of Iraq's more urbanized and stable regions, which likely facilitates greater access to healthcare and more consistent public health interventions than in other parts of the country. However, underreporting remains a significant challenge. Gaps in surveillance systems, limited diagnostic capacity in some health facilities, and inequitable access to care—especially in rural areas or among internally displaced individuals—can all contribute to an underestimation of the true burden of TB [23]. Moreover, because many regions rely primarily on passive case detection, individuals who are asymptomatic or exhibit only mild symptoms may go undiagnosed. These systemic limitations can lead to missed or delayed diagnoses, thereby sustaining community transmission and obscuring the true incidence of TB in the region.

**Figure 1:**
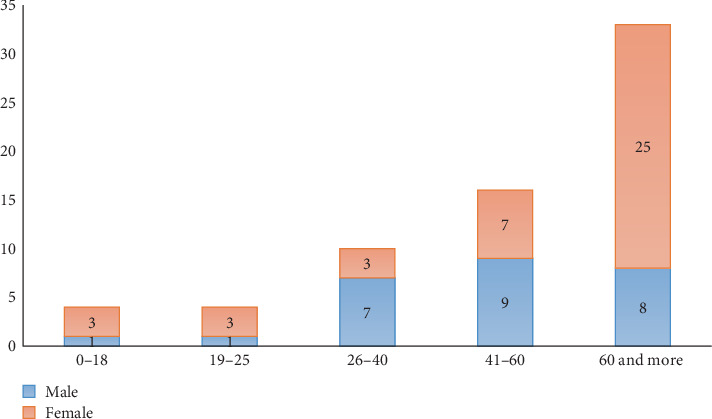
Age and gender distribution of pulmonary TB patients. The highest number of TB cases was observed in the ≥ 60-year age group, with a predominance of female patients.

Among the positive cases, the majority were female (*n* = 47, 61%), while 30 patients (39%) were male. Similarly, a study conducted in Erbil, Iraq, between 2012 and 2016 reported a higher percentage of female TB cases (*n* = 408, 56%) compared to males (*n* = 320, 44%) [22]. In contrast, a nearly equal male-to-female ratio of TB cases was reported in Sulaymaniyah in 2010, with 264 males and 266 females diagnosed [20]. Our data on gender distribution also contrasts global trends, where TB is typically more prevalent among males. According to the World Health Organization [23], men account for approximately 60%–70% of TB cases worldwide. The higher TB incidence in males is attributed to several risk factors, including smoking, occupational exposure, and delayed treatment seeking [24]. Conversely, the observed female predominance in our study may reflect regional differences in exposure, healthcare accessibility, or health-seeking behavior. In the Kurdistan Region of Iraq, women may have more frequent interactions with healthcare services—particularly in maternal and reproductive health contexts—potentially leading to earlier diagnosis and higher detection rates. Additionally, the demographic profile of the participating health facilities may have influenced the results, as they could serve a higher proportion of female patients. The age and gender distribution of the 67 confirmed TB patients is illustrated in Figure 1.

The majority of TB cases were in patients aged 60 years and older (42.9%), with a clear female predominance (25 females vs. 8 males). The relatively large proportion of TB cases among elderly individuals may contribute to the higher mortality observed in this demographic. This finding aligns with local epidemiological studies in Iraq, which report increased TB-related deaths among older adults [20, 25]. The female predominance in TB patients may be influenced by immunosenescence and comorbidities such as osteoporosis or diabetes, which can influence TB progression [26, 27]. This pattern is consistent with established epidemiological observations that immune function declines with age, increasing susceptibility to MTB infection in older individuals [28]. BCG vaccination, typically administered during infancy, provides protection for up to about 20 years [29]; booster doses beyond childhood are not routinely recommended, and revaccination has generally not shown significant benefit in adults [30]. Emerging evidence suggests that BCG vaccination in elderly individuals can modulate immune responses—enhancing memory T- and B-cell frequencies and potentially broadening nonspecific protection against respiratory infections—but whether this confers long-term protection against TB remains unclear [31]. In contrast, TB was more prevalent among men than women in the 26–40 age group (Figure 1). This may reflect occupational exposures and behavioral risk factors such as smoking and alcohol use, which are more common in men and increase susceptibility to TB [23, 32]. Biological sex differences in immune response may contribute, as testosterone can impair certain immune pathways, whereas estrogen appears to enhance protective responses against MTB [33].

**Figure 2:**
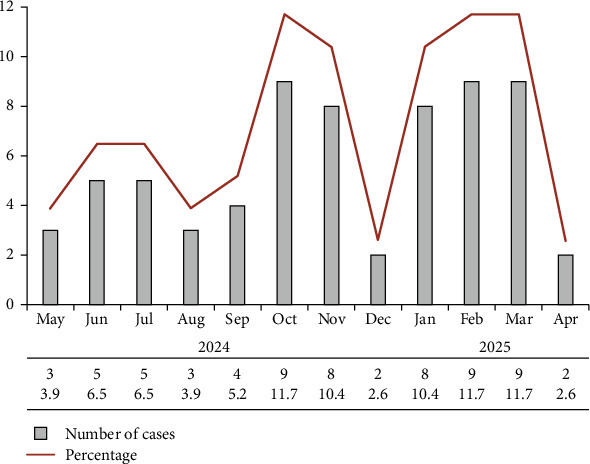
Monthly distribution of pulmonary TB cases in Sulaymaniyah, Iraq, from May 2024 to April 2025. The bar chart represents the number of TB cases each month, while the overlaid line indicates the corresponding percentage of cases relative to the total.

The monthly distribution of TB cases exhibited notable variation, with the highest number of cases observed between October and March (Figure 2), suggesting a potential seasonal pattern. This trend may be influenced by environmental factors affecting both disease transmission and host susceptibility. Previous studies have shown that variables such as temperature, humidity, and air quality can significantly impact TB incidence, often through effects on respiratory health and increased indoor crowding during colder months [34, 35]. These observations underscore the importance of intensified clinical vigilance and robust public health surveillance during specific seasonal windows. However, the lower number of cases reported in December may be explained by nonepidemiological factors, such as reduced healthcare service availability due to holiday-related closures and socioeconomic disruptions. Notably, in December 2024, several public sector institutions in Sulaymaniyah, including hospitals, experienced partial shutdowns or reduced working hours due to financial constraints and labor actions [36, 37].

### 2.8. Beijing and Non-Beijing Genotyping

**Figure 3:**
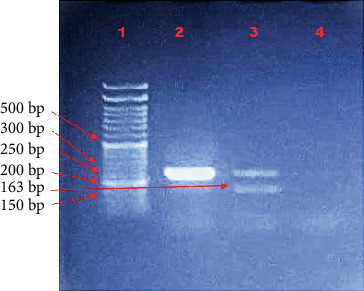
Multiplex PCR for differentiation between Beijing and non-Beijing strains. Lane 1: 50 bp ladder; Lane 2: 261 bp band representing the wild-type non-Beijing strain; Lane 3: 261 bp (non-Beijing strain) and 163 bp (Beijing strain); Lane 4: negative control.

Detection of MTB Beijing and non-Beijing strains in this study was performed by amplification of a specific region of the *Rv0679c* gene using multiplex PCR. This method, originally described by Nakajima et al. in 2013 [17], employs primer set consisting of one forward and two reverse primers. Differentiation between Beijing and non-Beijing strains was determined based on the size of the PCR amplicons: A 261 bp fragment indicated a non-Beijing strain, whereas a 163 bp fragment indicated a Beijing strain. These size differences arise from a single-nucleotide polymorphism (SNP) at Position 426 in the *Rv0679c* gene, where cytosine (C) is substituted by guanine (G), a mutation characteristic of Beijing lineage strains [17]. In this study, multiplex PCR followed by gel electrophoresis was performed on 50 out of the 77 DNA-extracted clinical samples (sputum and BAL). The results demonstrated that 49 samples (98.0%) were classified as non-Beijing strains, while 1 sample (2.0%) showed a mixed infection containing both Beijing and non-Beijing strains (Figure 3).

Further confirmation of the Beijing strain was obtained by performing two separate PCR reactions, each using the forward primer paired with a different reverse primer, followed by sequencing of the resulting amplicons. This analysis confirmed the Beijing-specific mutation. Although several international studies have reported the molecular detection of Beijing lineage strains, to our knowledge, no such lineage has previously been documented in Iraq. A related study performed in 2014 at Karbala University employed spoligotyping to detect MTB lineages among 70 MDR-MTB but did not identify any Beijing strains [38]. Therefore, this study provides the first molecular evidence of a Beijing strain in Sulaymaniyah, using a multiplex PCR-based approach.

The emergence of the Beijing lineage in Iraq may suggest cross-border transmission, potentially driven by population mobility, trade, or migration from neighboring countries where this strain is endemic, including Iran, China, and Central Asian republics [39, 40]. This lineage has been linked with increased virulence, higher rates of treatment failure, relapse, and a greater likelihood of developing MDR [41]. Given these clinical and epidemiological challenges, early molecular detection and continuous genotypic surveillance are critical to prevent wider dissemination [42]. Future studies should investigate the epidemiological origin, clinical impact, and resistance profiles of Beijing strains in Iraq to guide public health interventions and strengthen national TB control strategies.

### 2.9. Mutations in *rpoB* Gene

Total MTB genome was successfully extracted from 50 out of 77 microscopically positive samples. Conventional PCR was performed to amplify a 650 bp fragment of the *rpoB* gene within the RRDR. This amplicon spans Codons 507–533 and is a well-documented hotspot for mutations responsible for a large proportion of MRD TB cases [43, 44].

**Figure 4:**
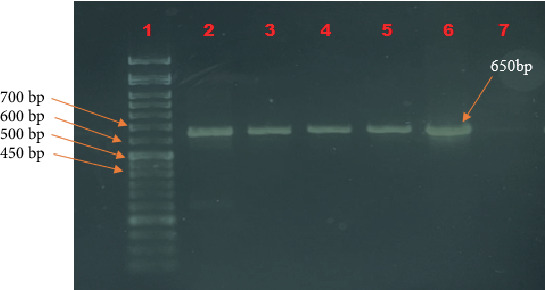
Gel electrophoresis of a 650 bp fragment of the *rpoB* gene. Lane 1: DNA ladder; Lanes 2–6: positive MTB samples of different strains; Lane 7: negative control.

Among the 50 clinical samples analyzed, 22 (44%) yielded positive PCR results, producing the expected 650 bp amplicon (Figure 4). Notably, sputum samples showed a slightly higher positivity rate (24%) compared to BAL samples (20%), suggesting that sputum may be a more suitable specimen type for direct molecular detection of MTB DNA in the absence of culture-based enrichment. This difference likely reflects the typically higher bacterial load in sputum, which more accurately represents the primary site of infection in pulmonary TB. In contrast, BAL specimens, although valuable for patients who are sputum-scarce or nonproductive, may contain fewer bacilli due to dilution by bronchoalveolar fluid and variation in collection technique, resulting in reduced PCR sensitivity [45].

Failure to amplify DNA in 56% of the samples may be attributed to several factors, including low mycobacterial DNA concentrations, degradation of nucleic acids during sample processing, or the presence of PCR inhibitors. The high rate of PCR-negative results, particularly among BAL samples, highlights the challenges of direct molecular detection from clinical specimens without prior culture [46]. Overall, these findings emphasize the importance of sample type and quality in molecular diagnostics of TB and support the use of sputum over BAL for direct PCR-based screening of rifampicin resistance via the *rpoB* gene.

DNA sequencing was performed for all 22 PCR-positive samples. Of these, six samples (12% of the total; 27.3% of PCR-positive samples) produced poor-quality chromatograms that were unsuitable for reliable analysis, potentially due to low DNA concentration, degraded template, or the presence of sequencing inhibitors. The remaining 16 samples (32% of the total; 72.7% of PCR-positive samples) yielded high-quality sequences, which were subsequently aligned and analyzed using BLAST against the wild-type MTB H37Rv reference strain.

All 16 *rpoB* gene nucleotide sequences obtained in this study were submitted to the GenBank database and assigned the following Accession Numbers: PV033318, PV033319, PV033320, PV033321, PV849998, PV849999, PV850000, PV850001, PV850002, PV850003, PV850004, PV850005, PV850006, PV850007, PV850008, and PV850009. Sequence analysis revealed that 14 of the 16 samples (28% of the total) exhibited no mutations within or outside the 81-bp RRDR, indicating wild-type *rpoB* sequences. The predominance of wild-type sequences (87.5%) suggests that most cases may remain susceptible to rifampicin in this region. In contrast, two samples (12.5% of the total positive sequencing samples), corresponding to Accession Numbers PV849998 and PV033321, harbored mutations within the sequenced products.

Further analysis revealed that the mutation observed in the sample with Accession Number PV849998 was located adjacent to the 81-bp RRDR, specifically at Codon 506. This mutation was synonymous (silent), with both codons encoding phenylalanine, and is therefore unlikely to contribute directly to rifampicin resistance. A previous study reported a nonsynonymous mutation at the same codon (TTC → TTA), resulting in an amino acid substitution from phenylalanine to leucine, which was associated with rifampicin resistance [47]. The *rpoB* gene sequence of sample PV033321 was particularly notable, exhibiting a total of 84 nucleotide substitutions. Among these, four mutations were identified within the 81-bp RRDR, specifically at Codons 523 (GGG → GGT), 524 (TTG → CTG), 528 (CGC → CGT), and 529 (CGA → CGC). All four were synonymous. In addition, 47 more synonymous mutations were observed upstream of this region, spanning Codons 534–714, as detailed in Table 2.

Although these mutations are synonymous and may not be directly linked to rifampicin resistance, their unusually high frequency among the isolates in this study suggests substantial genetic variability within the *rpoB* gene. This pattern may reflect ongoing microevolutionary processes in MTB, potentially driven by factors such as host immune pressure, environmental stress, or lineage-specific mutation rates [48, 49]. While silent mutations do not alter the amino acid sequence, they can still have functional consequences, for example, by affecting mRNA stability, translation efficiency, or protein folding [50]. Moreover, such mutations are increasingly recognized as valuable molecular markers for phylogenetic analysis, MTB strain differentiation, and tracking transmission dynamics [51, 52]. Based on these observations, we hypothesize that the accumulation of silent mutations may represent an early or intermediate stage in the evolutionary trajectory of MTB, potentially preceding the emergence of functionally significant, resistance-conferring mutations. This evolutionary trend underscores the importance of monitoring not only known resistance mutations but also neutral genetic changes that may serve as precursors or indicators of future adaptive shifts.

Moreover, several of the silent mutations identified in this study—particularly those within the RRDR of the *rpoB* gene—have also been reported in MTB isolates from various geographic regions. Notably, synonymous substitutions at Codons 514, 526, and 529 have been shown to cause false-positive results in widely used commercial molecular diagnostic assays, such as Xpert MTB/RIF and GenoType MTBDRplus, even in the absence of phenotypic resistance [53–55]. However, none of the strains reported in this study was classified as drug resistant based on the GeneXpert MTB/RIF results.

Additionally, 33 distinct nonsynonymous mutations were identified in sample PV033321, spanning Codons 600 to 719, each resulting in an amino acid substitution, including S600T, E602T, V604E, L606M, H614Y, and V615L (Table 3). These mutations were also located outside the canonical 81-bp RRDR [56]. Notably, none of the substitutions observed in this sample is reported in existing databases or prior studies, suggesting that they represent previously unreported (novel) *rpoB* mutations. While mutations outside the RRDR are rarely associated with rifampicin resistance, recent studies have documented their potential role in compensatory adaptations or low-level resistance phenotypes [57–59]. Further investigation, including phenotypic drug susceptibility testing and structural modeling, will be essential to determine whether any of these novel changes affect rifampicin binding or RNA polymerase function.

Attempts to obtain additional clinical information for this bacterial isolate were unsuccessful, as the elderly female patient from whom the sample was obtained had passed away, limiting further case investigation.

Although rifampicin-resistant MTB has been extensively studied worldwide, research on this topic in Iraq remains limited. Our investigation of *rpoB* gene mutations aligns closely with two previous studies conducted in Iraq. A 2023 study from Basra focused on detecting mutations within the 81-bp RRDR of clinical isolates and similarly reported a low prevalence of rifampicin-resistant strains [60]. A second study, conducted in Erbil in 2020, performed a molecular analysis of rifampicin-resistant MTB by examining mutations in the *rpoA*, *rpoB*, and *rpoC* genes and also reported a low frequency of rifampicin-resistant strains [61]. Our findings are generally consistent with these investigations; however, a key difference is that we detected no rifampicin-resistant MTB strains, a result corroborated by the GeneXpert assay. The silent mutations identified in our samples are likely undetectable by GeneXpert and do not confer resistance. Consequently, no resistant strains or functionally significant mutations were observed. This study has several limitations, including the relatively small number of bacterial isolates, the high PCR-negative rate, and the low proportion of positive sequencing results, which may restrict the generalizability of our findings. Furthermore, potential underreporting of TB cases in the city may have led to an underestimation of the true epidemiological burden. Despite these constraints, the study provides valuable insights into *rpoB* gene variability among local isolates and underscores the need for larger, more comprehensive investigations.

## 3. Conclusions

In summary, this study reveals a relatively low incidence of pulmonary TB in Sulaymaniyah city, alongside an unusual female predominance and a high burden among older adults, potentially influenced by waning vaccine protection and immunosenescence. Molecular analysis identified predominantly non-Beijing strains, with the first confirmed detection of the Beijing lineage in Iraq, highlighting potential regional shifts in TB epidemiology. Although no rifampicin-resistant mutations were detected in the *rpoB* gene, several novel synonymous and nonsynonymous mutations were documented, suggesting ongoing genetic evolution. These findings underscore the importance of enhanced molecular surveillance and targeted public health interventions to monitor emerging strains and prevent the spread of drug-resistant TB in the region.

## Figures and Tables

**Table 1:** List of primers with nucleotide sequences, amplicon sizes, and references.

**Gene name**	**Primer sequence**	**Product size**	**Reference**
*Rv0679c*	FW: GTCACTGAACGTGGCCGGCTC		[17]
	RI: TCGGTCACCGTTTTTGTAGGTGACCGTC	163 bp	
	RII: AGCAACCTCGCAATCTGACC	261 bp	
*rpoB*	Forward: GGTGGAAACCGACGACATC	650 bp	This study
	Reverse: CGTCCATGTAGTCCACCTCA		

**Table 2:** Synonymous mutations in the *rpoB* gene of MTB sample PV033321.

***rpoB* codon number**	**MTB H37RV codon sequence**	**MTB sample PV033321 codon sequence**	**Amino acids**
523	GGG	GGT	Glycine
524	TTG	CTG	Leucine
528	CGC	CGT	Arginine
529	CGA	CGC	Arginine
534	GGG	GGC	Glycine
535	CCC	CCG	Proline
539	TCA	AGC	Serine
544	GGG	GGC	Glycine
545	CTG	CTC	Leucine
547	GTC	GTG	Valine
552	CCG	CCC	Proline
553	TCG	ACG	Serine
557	CGG	CGC	Arginine
562	GAA	GAG	Glutamic acid
564	CCT	CCG	Proline
565	GAG	GAA	Glutamic acid
567	CCC	CCG	Proline
588	GAA	GAG	Glutamic acid
589	ACG	ACC	Threonine
591	TAC	TAT	Tyrosine
594	GTG	GTC	Valine
598	GTG	GTC	Valine
599	GTT	GTC	Valine
612	GAC	GAT	Aspartic acid
613	CGC	CGT	Arginine
620	AAT	AAC	Asparagine
624	GAT	GAC	Aspartic acid
627	GGT	GGC	Glycine
634	GTG	GTC	Valine
651	GTG	GTC	Valine
662	GTG	GTC	Valine
664	GTG	GTC	Valine
665	GCC	GCG	Alanine
669	ATT	ATC	Isoleucine
672	CTG	CTC	Leucine
679	CGT	CGC	Arginine
681	CTC	CTG	Leucine
683	GGG	GGT	Glycine
684	GCA	GCG	Alanine
688	CGC	CGT	Arginine
690	GCG	GCC	Alanine
694	GTC	GTG	Valine
695	CGT	CGC	Arginine
699	CCG	CCT	Proline
701	GTG	GTC	Valine
704	GGG	GGC	Glycine
707	CTG	TTG	Leucine
708	CGC	CGT	Arginine
709	GCG	GCC	Alanine
710	GCG	GCC	Alanine
714	GGC	GGT	Glutamine

**Table 3:** Nonsynonymous mutations in the *rpoB* gene of MTB sample PV033321.

***rpoB* codon number**	**MTB H37RV nucleotides (wild type)**	**MTB strain PV033321**	**Amino acid changes**
600	AGC	ACC	S600T
602	GAG	ACC	E602T
604	GTG	GAG	V604E
606	CTG	ATG	L606M
614	CAC	TAC	H614Y
615	GTG	CTC	V615L
616	GTG	ATC	V616I
617	GCA	GGT	A617G
621	TCG	ACC	S621T
622	CCG	AGC	P622S
623	ATC	TAC	I623Y
625	GCG	CTG	A625L
626	GAC	AGC	D626S
628	CGC	AAC	R628N
629	TTC	ATC	F629I
630	GTC	ACC	V630T
631	GAG	GAC	E631D
632	CCG	GAG	P632E
638	CGC	AAG	R638K
640	GCG	GGC	A640G
641	GGC	TCC	G641S
645	TAC	TTC	Y645F
647	CCC	GAC	P647D
648	TCG	GCG	S648A
649	TCT	GCG	S649A
650	GAG	CAG	E650Q
652	GAC	GAG	D652E
654	ATG	CTC	M654L
696	AGC	AAC	S696N
698	ACC	TCG	A698S
711	ATC	GTC	I711V
717	GTC	ATC	V717I
719	GCC	TCG	A719S

## Data Availability

The data that support the findings of this study are available from the corresponding author upon reasonable request. All relevant experimental details are included within the manuscript.
